# Supplementary material for: Room-temperature intrinsic ferromagnetism in epitaxial CrTe2 ultrathin films
Source: Nat Commun. 2021 May 3;12:2492. doi: 10.1038/s41467-021-22777-x (PMC8093203; doi:10.1038/s41467-021-22777-x)
Supplement: Supplementary file 1 — Supplementary Information [file 41467_2021_22777_MOESM1_ESM.pdf]

Supplementary Materials

Room-temperature intrinsic ferromagnetism in epitaxial $CrTe_2$ ultrathin films

Xiaoqian Zhang,[1,2#] Qiangsheng Lu,[2#] Wenqing Liu,[1,3#] Wei Niu,[4] Jiabao Sun,[3] Jacob Cook,[2] Mitchel Vaninger,[2] Paul. F. Miceli,[2] David J. Singh,[2,5] Shang-Wei Lian,[6] Tay-Rong Chang,[6,7] Xiaoqing He,[8,9] Jun Du,[10] Liang He,[1*] Rong Zhang,[1*] Guang Bian,[2*] Yongbing Xu[1,11*]

[1]Jiangsu Provincial Key Laboratory of Advanced Photonic and Electronic Materials, School of Electronic Science and Engineering, Nanjing University, Nanjing 210093, China

[2]Department of Physics and Astronomy, University of Missouri, Columbia, Missouri 65211, USA

[3]Department of Electronic Engineering, Royal Holloway University of London, Egham, Surrey TW20 0EX, UK

[4]New Energy Technology Engineering Laboratory of Jiangsu Provence & School of Science, Nanjing University of Posts and Telecommunications, Nanjing 210023, China

[5]Department of Chemistry, University of Missouri, Columbia, Missouri 65211, USA

[6]Department of Physics, National Cheng Kung University, Tainan 701, Taiwan

[7]Center for Quantum Frontiers of Research and Technology (QFort), Tainan 701, Taiwan

[8]Electron Microscopy Core Facility, University of Missouri, Columbia, Missouri 65211, USA

[9]Department of Mechanical and Aerospace Engineering, University of Missouri, Columbia, MO 65211, USA

[10]National Laboratory of Solid State Microstructures and Department of Physics, Nanjing University, Nanjing 210093, China

[11]York-Nanjing Joint Centre (YNJC) for spintronics and nano engineering, Department of Electronic Engineering, The University of York, YO10 3DD, United Kingdom

[#]These authors contribute equally to this work.

*Correspondence and requests for materials should be addressed to Y.X. (ybxu@nju.edu.cn), G.B. (biang@missouri.edu), L.H. (heliang@nju.edu.cn), R.Z. (rzhang@nju.edu.cn)

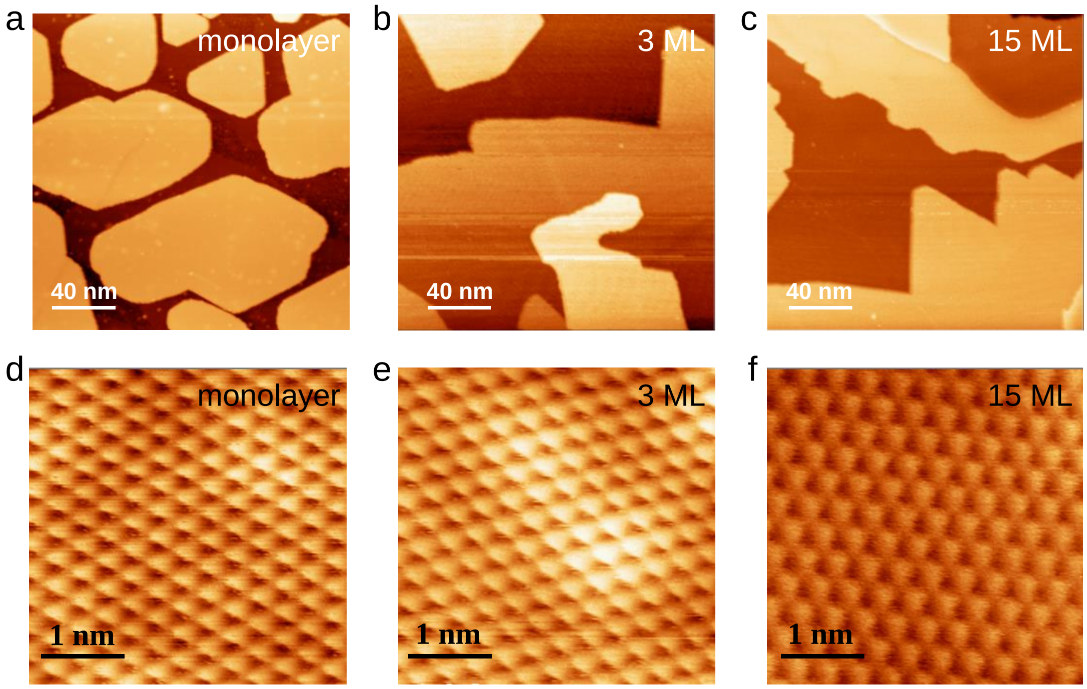

Supplementary Figure 1 | STM images of $CrTe_2$ thin films with different thicknesses. a-c, Surface morphology of uniform (**a**) monolayer, (**b**) 3 ML and (**c**) 15 ML $CrTe_2$ thin films. **d-f**, Atomically resolved STM images of the corresponding as-grown $CrTe_2$ thin films with a hexagonal structure in (**a-c**).

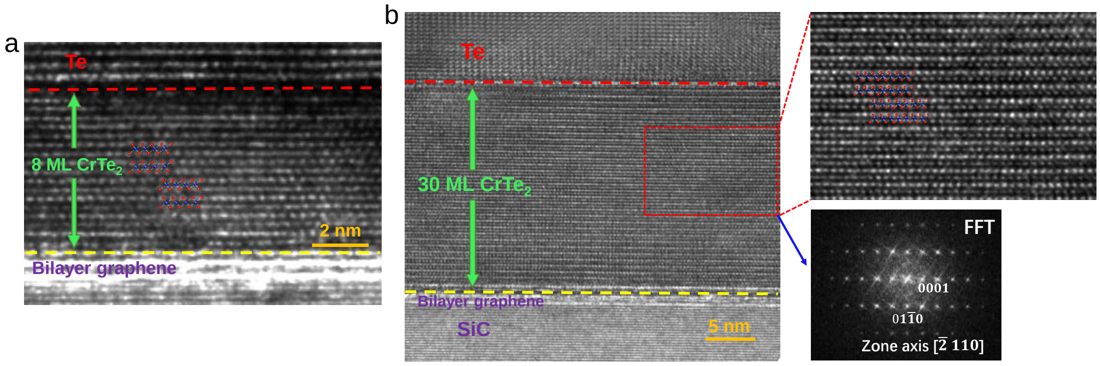

Supplementary Figure 2 | Atomic-resolution HAADF-STEM images of $CrTe_2$ thin films with different thicknesses. The inset atomic model demonstrates the lattice structure of 1T-$CrTe_2$ in cross-sectional view. Cr atoms are fairly light compared with Te, leading to a quite low intensity contribution[1,2].

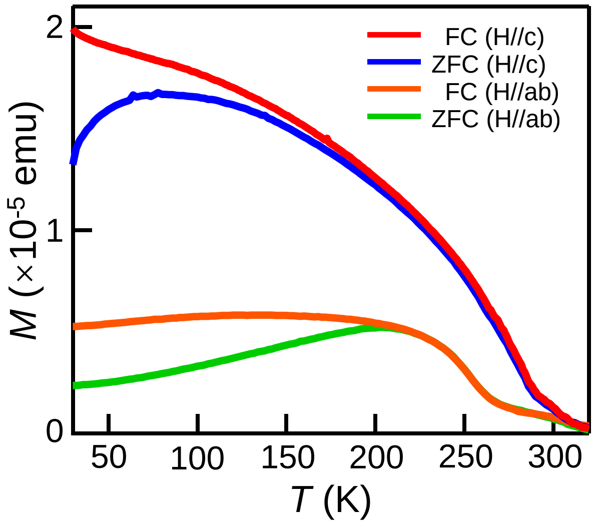

Supplementary Figure 3 | Zero-field and field cooled temperature dependence of the magnetization of 7 ML $CrTe_2$ with an applied in-plane and out-of-plane magnetic field of 0.1 T. The out-of-plane magnetization increases with decreasing temperature, indicating the enhanced ferromagnetism at low temperature. On the contrary, the slight decrease of in-plane magnetization at low temperature is resulted from the accelerated process of spin reorientation from the *ab* plane to the *c* axis[3], due to the weak in-plane magnetic field.

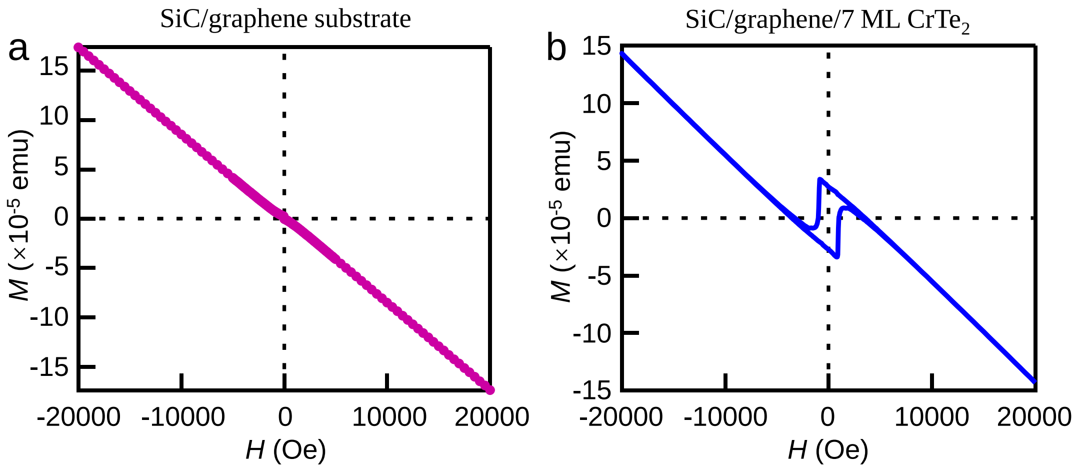

Supplementary Figure 4 | Eliminating the possible magnetic contribution from the substrate. a, The out-of-plane field dependent magnetization of SiC/graphene substrate taken at 20 K shows a linear relationship, demonstrating a typical diamagnetic behavior. **b**, Well-defined magnetic hysteresis loops of 7 ML $CrTe_2$ film without subtracting the diamagnetic background from substrate, indicating the intrinsic ferromagnetism of $CrTe_2$ thin films.

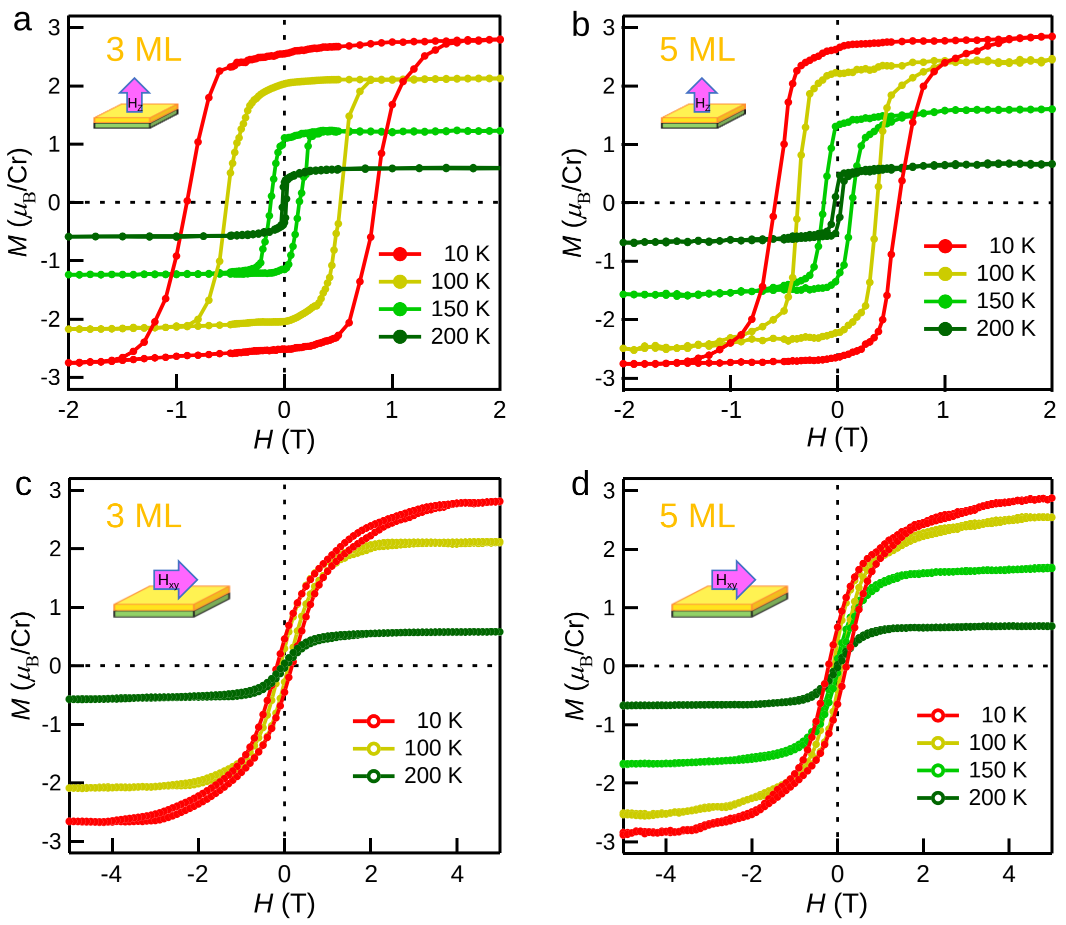

Supplementary Figure 5 | M-H loops of $CrTe_2$ thin films with different thicknesses. a-d, Temperature dependent magnetic hysteresis loops of (**a**,**c**) 3 ML and (**b**,**d**) 5 ML $CrTe_2$ thin films with out-of-plane and in-plane field, indicating a strong out-of-plane magnetic anisotropy. At 10 K, the magnetic moments of 3 ML and 5 ML $CrTe_2$ are found to be 2.81 μ_B/Cr and 2.83 μ_B/Cr, respectively, which are comparable with the theoretical value (~3 μ_B/Cr)[4]. With in-plane and out-of-plane **M-H** loops, the PMA constants of 3 ML and 5 ML $CrTe_2$ films are determined to be 6.6×10^6 erg/cm^3 and 6.5×10^6 erg/cm^3 at 10 K, respectively.

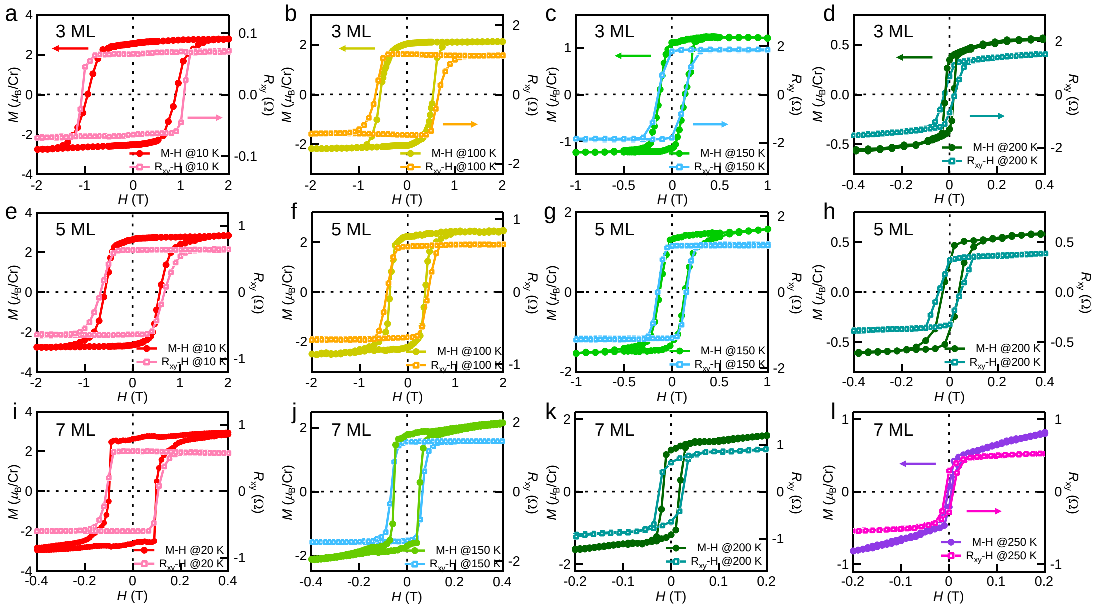

Supplementary Figure 6 | Electrical transport and magnetic measurements of $CrTe_2$ thin films. The field dependent Hall resistance of $CrTe_2$ thin films with thickness of (**a-d**) 3 ML, (**e-f**) 5 ML and (**i-l**) 7 ML at various temperatures share a similar trend with the magnetic hysteresis loops.

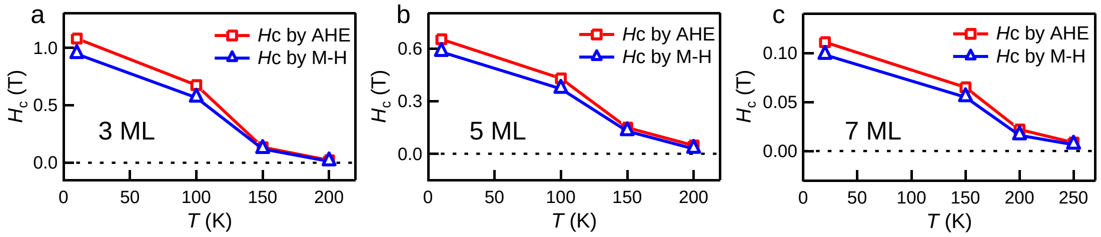

Supplementary Figure 7 | Comparison of coercivities measured by electrical transport and magnetic measurements. The coercive fields extracted from the Hall responses and **M-H** loops are consistent with each other.

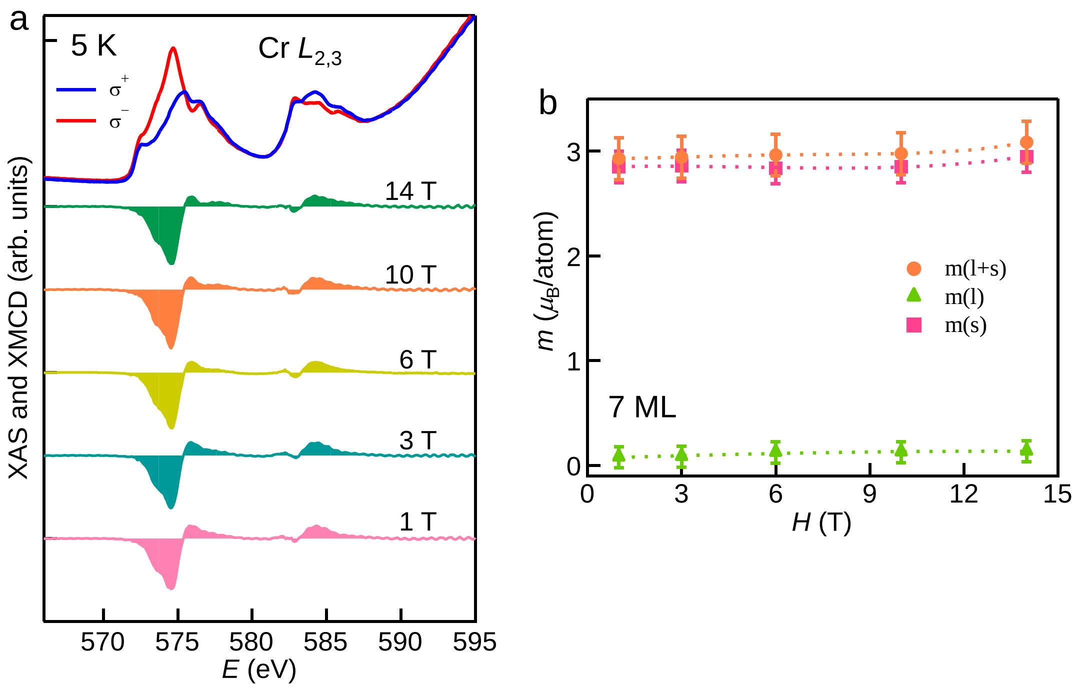

Supplementary Figure 8 | **Field dependent XMCD characterization. a**, The XMCD line shape of the $CrTe_2$ thin film is invariable with the increasing field. **b**, Field dependences of m_{l+s}, m_s and m_l by sum rules. The m_s and m_l show little variation with the magnetic field ranging from 1 to 14 T. The error bars reflect the uncertainties in the background estimation for the XMCD sum rules analysis. To exclude the presence of paramagnetism, we repeated the experiment at enhanced magnetic fields up to 14 T. A rather constant m_s and m_l is observed at saturation from 1 T to 14 T along out-of-plane direction within error bars, ruling out the possibility of paramagnetic contribution[5].

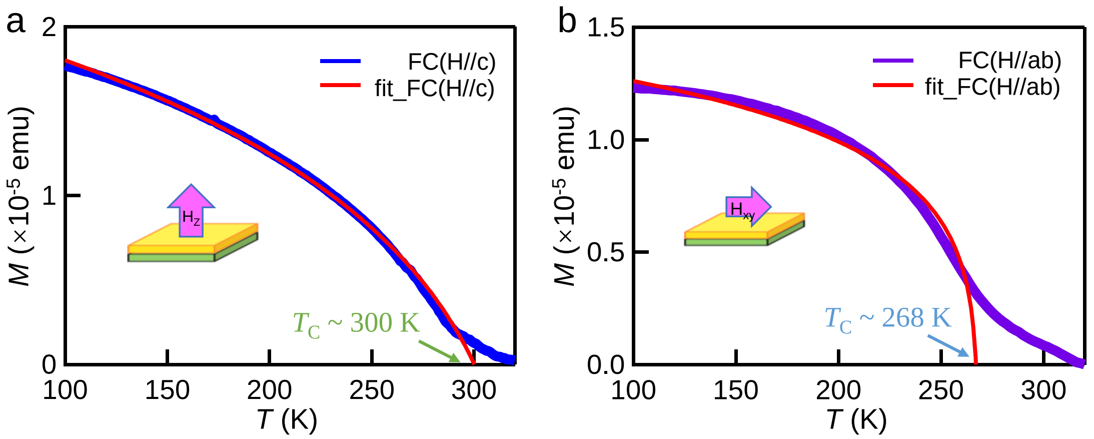

Supplementary Figure 9 | Criticality analysis for 7 ML $CrTe_2$ thin films with out-of-plane (0.1 T) and in-plane magnetic field (0.5 T). The solid lines are the fitting curves with the form of $\alpha(1-T/T_C)^{\beta}$. The T_C of 7 ML $CrTe_2$ thin films extracted from the temperature-dependent out-of-plane and in-plane magnetization are ~300±5 K and 268±3 K, respectively[6]. The T_C calculated along the hard axis is lower than that of the easy axis. The theoretical work by Callen[7] shows that for an anisotropic ferromagnet, the T_C depends on the direction of the magnetization for an anisotropy energy comparable to the exchange energy. Distinct with 3D materials in which the typical value of exchange interaction is orders of magnitudes larger than magnetic anisotropy, the T_C in 2D ferromagnets is determined primarily by the excitation gap that results from the magnetic anisotropy[8]. Therefore, the dominant magnetic anisotropy in 2D magnetic materials gives rise to the variation of T_C along different magnetic directions. Similar behaviors were also observed in Fe_3GeTe_2 (201 K along the easy axis and 196 K along the hard axis)[9] and Fe_7S_8 single crystals (603 K along the easy axis and 225 K along the hard axis)[10].

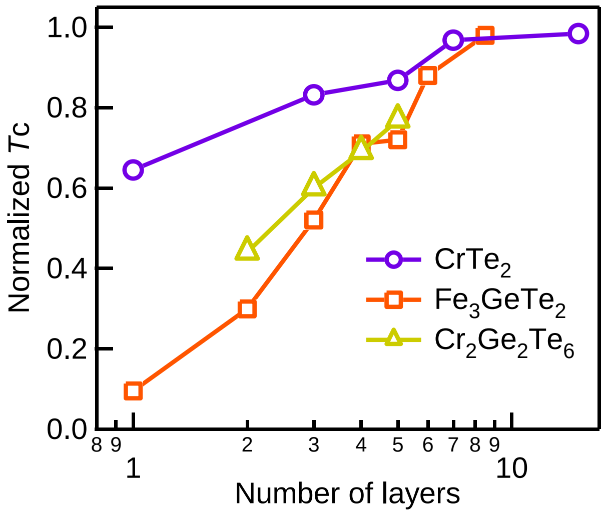

Supplementary Figure 10 | T_C (normalized to bulk T_C for the particular material) as a function of the number of layers. Data for Fe_3GeTe_2 adapted from ref. [11]; for $Cr_2Ge_2Te_6$, ref. [8].

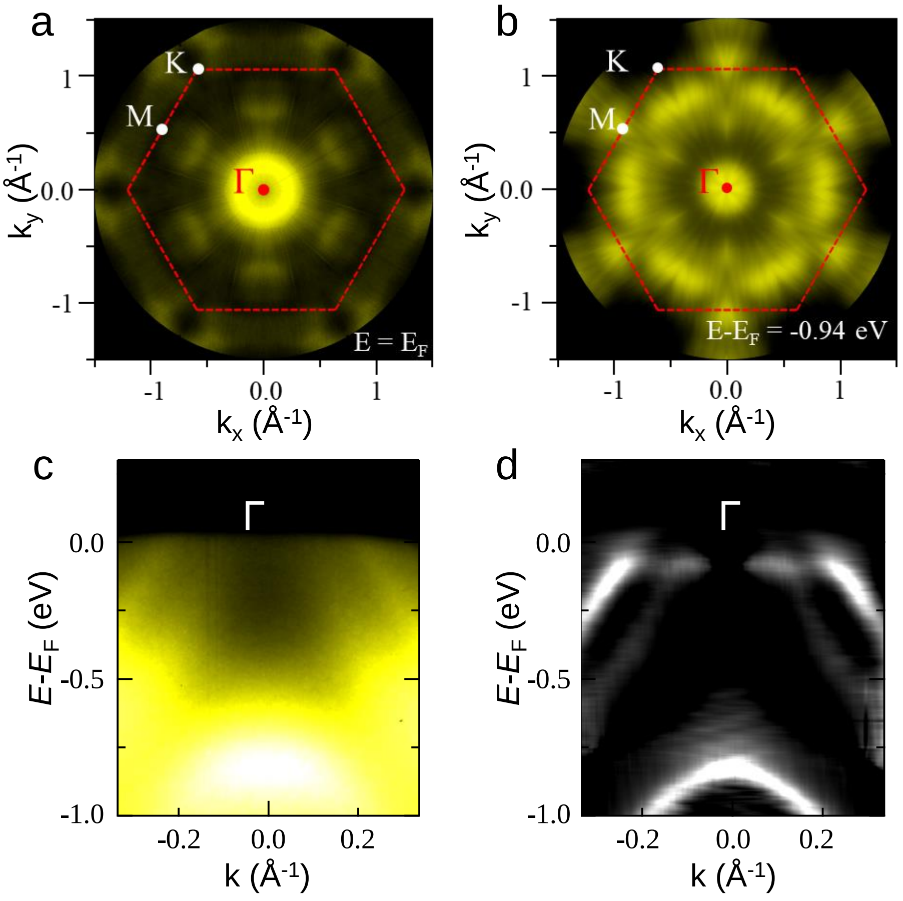

Supplementary Figure 11 | Fermi surface mapping and hole pockets near E_F. **a**,**b**, ARPES-intensity mapping at the (**a**) Fermi level and (**b**) binding energy of 0.94 eV as a function of two-dimensional wavevector measured by photon energy of 21.2 eV. The hexagonal Brillouin zone (red dashed lines) and high-symmetry points (white dots) are marked. The Fermi surface consists of two concentric cone-like bands contributed by the hole pockets. **c**,**d**, The (**c**) magnified portions and (**d**) second-derivative plot of few-layer $CrTe_2$ near E_F with enhanced contrast measured by photon energy of 21.2 eV, in which the large hole pockets are centered at Γ point.

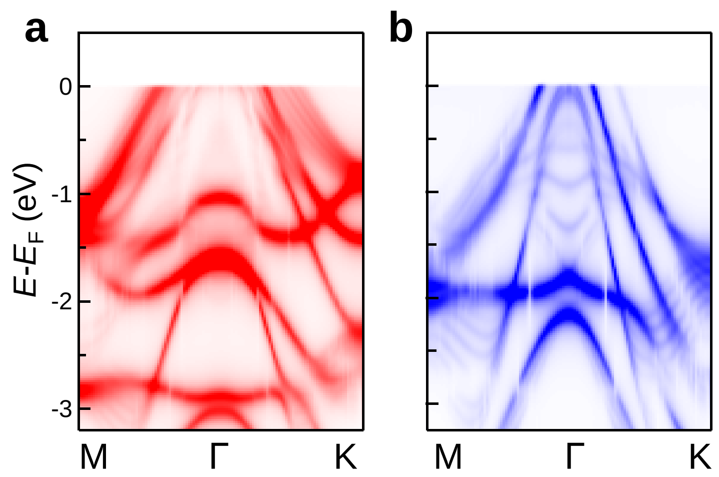

Supplementary Figure 12 | Band structure by DFT calculations. a,**b**, The images of calculated (**a**) minority and (**b**) majority spin projections of the bands. To better illustrate the spin contribution to electronic structure, the calculated minority band and majority band are separately plotted.

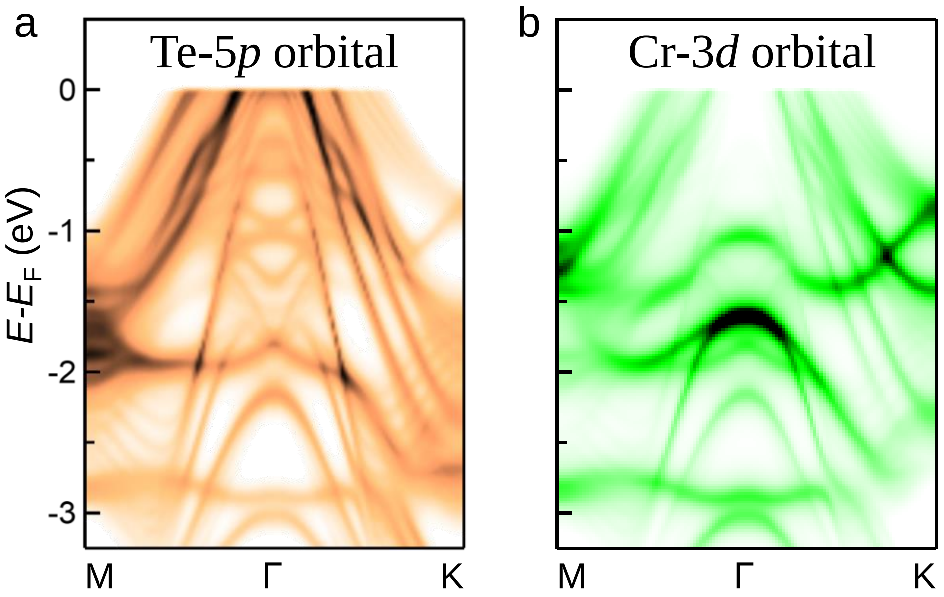

Supplementary Figure 13 | Orbital and surface projection analysis of the band structure. a,**b**, The calculated $CrTe_2$ band structure of (**a**) Te-5*p* and (**b**) Cr-3*d* orbital character. Higher intensity in the picture indicates the stronger surface-projected charge density.

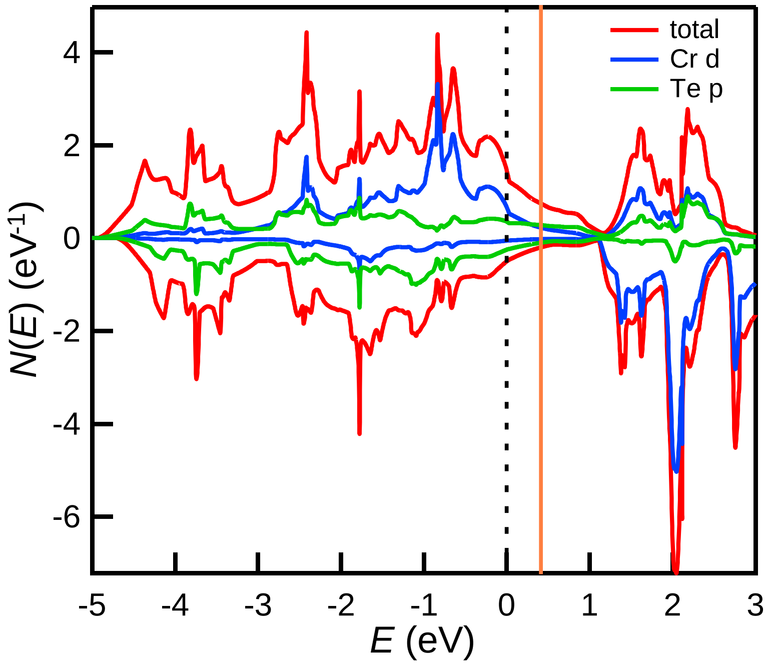

Supplementary Figure 14 | Calculated density of states of bulk $CrTe_2$. According to the DFT calculations, the hybridization of Cr 3*d* and Te 5*p* states is confirmed. A notable difference is the position of the theoretical E_F (black dashed lines) and experimental E_F (orange solid lines), indicating an n-type doping of $CrTe_2$.

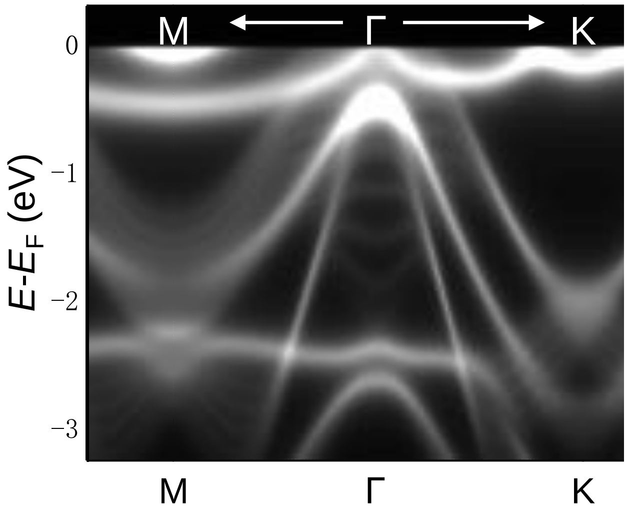

Supplementary Figure 15 | First-principles calculations of the band structures without the inclusion of spin polarization. Comparison between the calculated bands with experimental ARPES dispersions confirms that calculations including spin polarization fit much better.

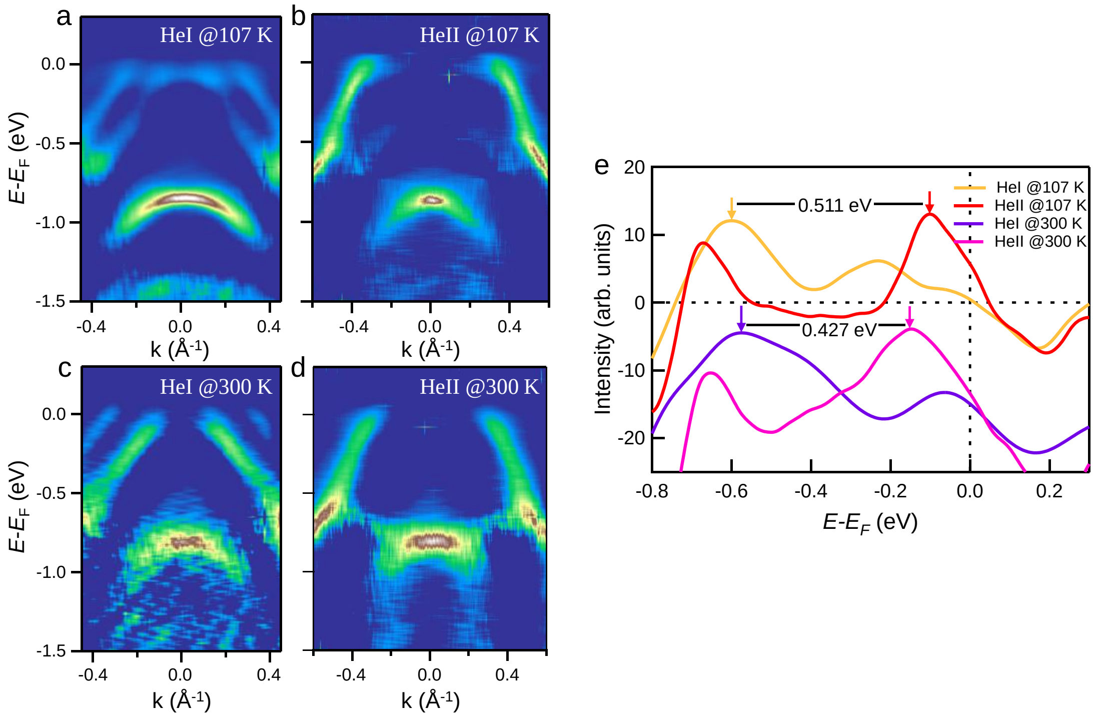

Supplementary Figure 16 | Temperature dependent spin splitting. a-d, Temperature evolution of second-derivative valence structure near the E_F taken by (**a**,**c**) He Iα (21.2 eV), (**b**,**d**) He IIα photons (40.8 eV) along the high symmetry direction M-Γ-M. **e**, energy distribution curves (EDCs) of panel (**a-d**) at k = -0.36 $Å^{-1}$. Peak positions of hole bands are indicated by the arrows. The energy splitting value could be obtained from the temperature dependent EDCs of the second derivative data. The fitted peak-to-peak splitting of majority and minority bands at 300 K shows an obvious decreasing trend compared with 107 K, corresponding to the weaker ferromagnetism at higher temperature.

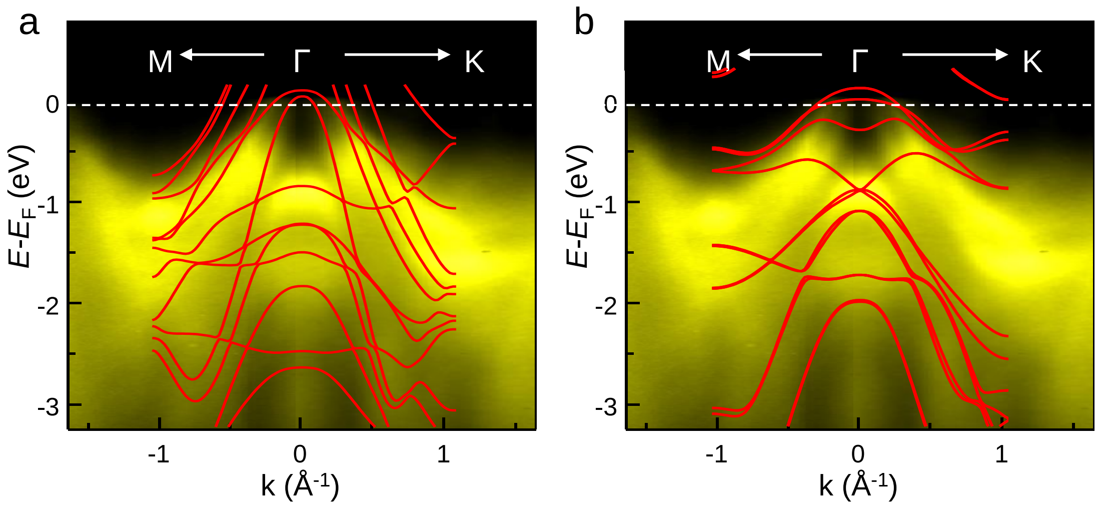

Supplementary Figure 17 | Comparison among experimental band dispersion and DFT calculations. a,**b**, Calculated band structure obtained from the first-principles band calculations for (**a**) 1T and (**b**) 2H phases compared with the ARPES-intensity plot of 7 ML $CrTe_2$. The bands of 2H phase show two main features including the flat hole-like bands near Γ and a "M"-shape flat band with a top edge at binding energy (E_B) of ~0.6 eV. Those features are absent in the ARPES-spectra. On the contrary, the ARPES-intensity plots and calculated band structure for 1T-$CrTe_2$ share most common features. For example, the hole-like bands crossing the E_F around Γ point and a relatively flat Cr 3*d* orbital band at E_B of ~1 eV. These characteristic bands establish the intrinsic differences between the electronic states of the 1T and 2H phases, and further confirm the trigonal phase of $CrTe_2$ thin films.

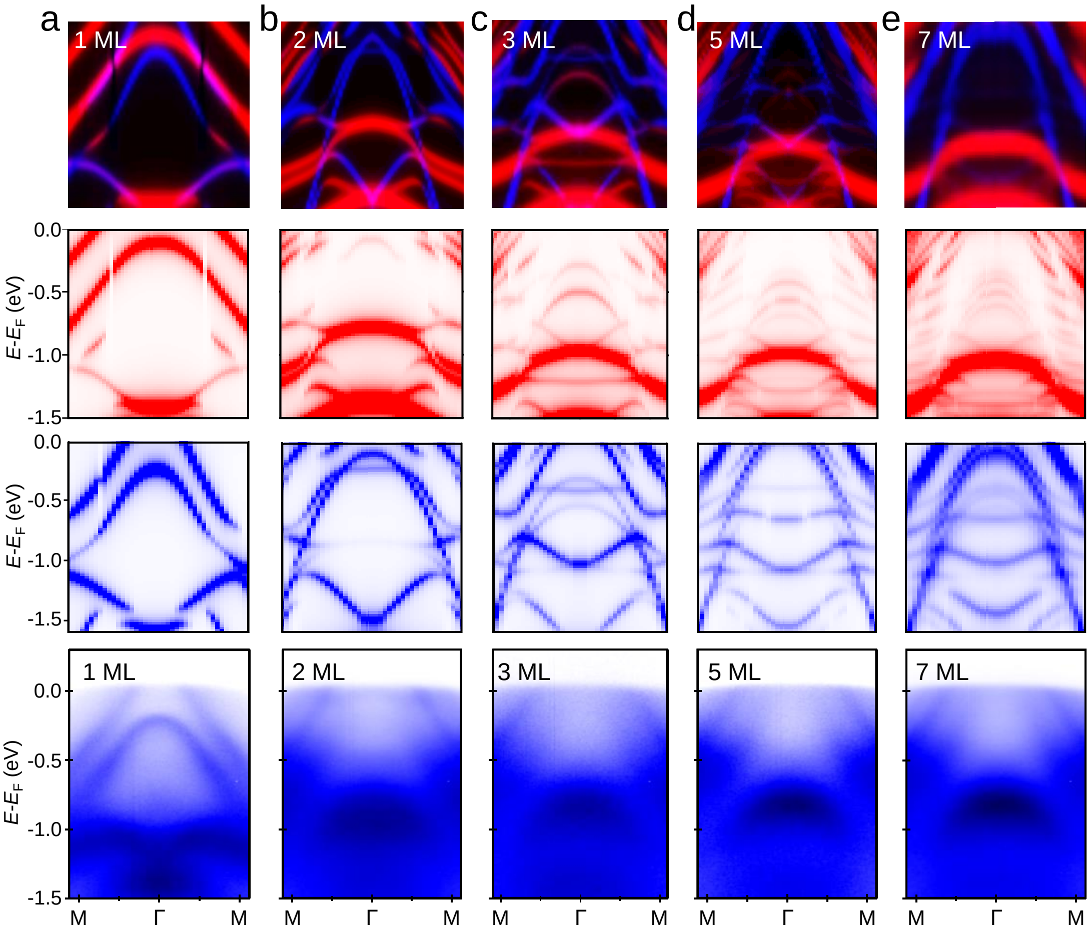

Supplementary Figure 18 | Band structures of $CrTe_2$ ultrathin films. a, 1 ML. **b**, 2 ML. **c**, 3 ML. **d**, 5 ML. **e**, 7 ML. All the spectra were taken along the high symmetry direction M-Γ-M. Upper panels: band structures of 1T phase from first-principles calculations; Middle panels: calculated minority (red) and majority (blue) spin projections of the bands, respectively; Lower panels: ARPES intensity maps.

Material	Maximal K_u (Merg/cm^3)	Ref.
**7 ML $CrTe_2$ films**	**5.63**	**[*]**
Fe_3GeTe_2 nanoflakes	1.5	[12]
CrI_3 bulk	3	[13]
$Cr_2Ge_2Te_6$ bulk	0.48	[14]
Fe_2CrSi films	2.8	[15]
$[Co/Pd]_6$ films	3.4	[16]
$[Co/Pt]_6$ films	9	[16]
$L0_{22}$-Mn_3Ga films	0.89	[17]
CoFeB films	2.1	[18]
Co_2FeAl films	1.3	[19]

Supplementary Table 1 | Maximal K_u in various strong PMA systems from literatures. The K_u in $CrTe_2$ film system is comparable to the typical values in famous ferromagnetic systems with strong PMA, which is of crucial importance to maintain high thermal stability at moderate coercive fields for long-term data retention in high-density magnetic data-storage devices.

References

1 Lazar, S. *et al.* Imaging, core-loss, and low-loss electron-energy-loss spectroscopy mapping in aberration-corrected STEM. *Microsc. Microanal.* **16**, 416-424 (2010).

2 Chen, J. *et al.* Evidence for Magnetic Skyrmions at the Interface of Ferromagnet/Topological-Insulator Heterostructures. *Nano Lett.* **19**, 6144-6151 (2019).

3 Wang, Q., Sun, S., Zhang, X., Pang, F. & Lei, H. Anomalous Hall effect in a ferromagnetic Fe_3Sn_2 single crystal with a geometrically frustrated Fe bilayer kagome lattice. *Phys. Rev. B* **94**, 075135 (2016).

4 Otero Fumega, A., Phillips, J. & Pardo, V. Controlled Two-Dimensional Ferromagnetism in 1T–$CrTe_2$: The Role of Charge Density Wave and Strain. *J. Phys. Chem. C* **124**, 21047-21053 (2020).

5 Liu, W. Q. *et al.* Spin and orbital moments of nanoscale Fe3O4 epitaxial thin film on MgO/GaAs(100). *Appl. Phys. Lett.* **104**, 142407 (2014).

6 Fei, Z. *et al.* Two-dimensional itinerant ferromagnetism in atomically thin Fe_3GeTe_2. *Nat. Mater.* **17**, 778-782 (2018).

7 Callen, E. R. Anisotropic Curie Temperature. *Phys. Rev.* **124**, 1373-1379 (1961).

8 Gong, C. *et al.* Discovery of intrinsic ferromagnetism in two-dimensional van der Waals crystals. *Nature* **546**, 265-269 (2017).

9 Wang, Y. *et al.* Anisotropic anomalous Hall effect in triangular itinerant ferromagnet Fe_3GeTe_2. *Phys. Rev. B* **96**, 134428 (2017).

10 Armstrong, J. N., Hua, S. Z. & Chopra, H. D. Anisotropic Curie temperature materials. *Phys. Status Solidi B* **250**, 387-395 (2013).

11 Deng, Y. *et al.* Gate-tunable room-temperature ferromagnetism in two-dimensional Fe_3GeTe_2. *Nature* **563**, 94-99 (2018).

12 Kim, D. *et al.* Antiferromagnetic coupling of van der Waals ferromagnetic Fe_3GeTe_2. *Nanotechnology* **30**, 245701 (2019).

13 Richter, N. *et al.* Temperature-dependent magnetic anisotropy in the layered magnetic semiconductors CrI_3 and $CrBr_3$. *Phys. Rev. Mater.* **2**, 024004 (2018).

14 Zeisner, J. *et al.* Magnetic anisotropy and spin-polarized two-dimensional electron gas in the van der Waals ferromagnet $Cr_2Ge_2Te_6$. *Phys. Rev. B* **99**, 165109 (2019).

15 Wang, Y.-P. *et al.* Perpendicular magnetic anisotropy in $Fe_2Cr_{1-x}Co_xSi$ Heusler alloy. *J. Phys. D: Appl. Phys.* **47**, 495002 (2014).

16 Yakushiji, K. *et al.* Ultrathin Co/Pt and Co/Pd superlattice films for MgO-based perpendicular magnetic tunnel junctions. *Appl. Phys. Lett.* **97**, 232508 (2010).

17 Kurt, H., Rode, K., Venkatesan, M., Stamenov, P. & Coey, J. M. D. High spin polarization in epitaxial films of ferrimagnetic Mn_3Ga. *Phys. Rev. B* **83**, 020405 (2011).

18 Ikeda, S. *et al.* A perpendicular-anisotropy CoFeB-MgO magnetic tunnel junction. *Nat. Mater.* **9**, 721-724 (2010).

19 Wu, Y., Xu, X. G., Miao, J. & Jiang, Y. Perpendicular Magnetic Anisotropy in Co-Based Full Heusler Alloy Thin Films. *Spin* **5**, 1540012 (2016).
